# Personalized Medicine in Cancer Pain Management

**DOI:** 10.3390/jpm13081201

**Published:** 2023-07-28

**Authors:** Mohammad Raad, William Omar Contreras López, Alireza Sharafshah, Marjan Assefi, Kai-Uwe Lewandrowski

**Affiliations:** 1 Department of Molecular, Cellular and Biomedical Sciences, University of New Hampshire, Durham, NH 03824, USA; 2 Neurosurgeon Clinica Foscal Internacional, Bucaramanga 680006, Colombia; wyllcon@gmail.com; 3 Neurosurgeon Clinica Portoazul, Caribe, La Merced, Asunción, Centro, Barranquilla 680006, Colombia; 4 Cellular and Molecular Research Center, School of Medicine, Guilan University of Medical Sciences, Rasht 41937-1311, Iran; alirezasharafshah@yahoo.com; 5 University of North Carolina, Greensboro, NC 27412, USA; massefi@aggies.ncat.edu; 6 Center for Advanced Spine Care of Southern Arizona, Tucson, AZ 85712, USA; business@tucsonspine.com; 7 Department of Orthopaedics, Fundación Universitaria Sanitas, Bogotá 111321, Colombia; 8 Department of Orthopedics, Hospital Universitário Gaffre e Guinle, Universidade Federal do Estado do Rio de Janeiro, Rio de Janeiro 20270-004, Brazil

**Keywords:** cancer, pain, personalized medicine, pharmacogenomics, cancer pain management, variant

## Abstract

Background: Previous studies have documented pain as an important concern for quality of life (QoL) and one of the most challenging manifestations for cancer patients. Thus, cancer pain management (CPM) plays a key role in treating pain related to cancer. The aim of this systematic review was to investigate CPM, with an emphasis on personalized medicine, and introduce new pharmacogenomics-based procedures for detecting and treating cancer pain patients. Methods: This study systematically reviewed PubMed from 1990 to 2023 using keywords such as cancer, pain, and personalized medicine. A total of 597 publications were found, and after multiple filtering processes, 75 papers were included. In silico analyses were performed using the GeneCards, STRING-MODEL, miRTargetLink2, and PharmGKB databases. Results: The results reveal that recent reports have mainly focused on personalized medicine strategies for CPM, and pharmacogenomics-based data are rapidly being introduced. The literature review of the 75 highly relevant publications, combined with the bioinformatics results, identified a list of 57 evidence-based genes as the primary gene list for further personalized medicine approaches. The most frequently mentioned genes were CYP2D6, COMT, and OPRM1. Moreover, among the 127 variants identified through both the literature review and data mining in the PharmGKB database, 21 variants remain as potential candidates for whole-exome sequencing (WES) analysis. Interestingly, hsa-miR-34a-5p and hsa-miR-146a-5p were suggested as putative circulating biomarkers for cancer pain prognosis and diagnosis. Conclusions: In conclusion, this study highlights personalized medicine as the most promising strategy in CPM, utilizing pharmacogenomics-based approaches to alleviate cancer pain.

## 1. Introduction

Cancer pain is an important healthcare and quality of life (QoL) concern [1]. One of the most prevalent, challenging, and unpleasant manifestations that cancer patients experience is pain. A meta-analysis that investigated 122 studies with 4199 subjects found that around 55% of cancer patients endure pain throughout therapy, with 40% experiencing pain following curative treatment. Cancer patients may have pain due to cancer itself, including bone metastases, cancer penetrating soft tissues, or nerve compression [2]. Cancer patients may also have acute pain from the side effects of cancer treatment, including mucositis caused by chemotherapy, musculoskeletal pain caused by chemotherapy, post-operative wound pain, mucositis caused by radiation, enteritis, or dermatitis [3,4,5,6,7]. As a long-term side effect of cancer treatment, the pain might become chronic for several cancer survivors [8].

Pharmacologic and non-pharmacologic therapies are used to treat cancer pain management (CPM) [9]. Opioids are the gold standard for treating cancer-related pain that ranges from moderate to severe. Sufficient pain alleviation in different cancer pain syndromes is frequently found when combined with additional adjuvant therapies [10]. Opioids including oxycodone, morphine, hydromorphone, fentanyl, are essential in controlling somatic pain, and anticonvulsants, such as gabapentin and pregabalin, are among the pharmacologic options for neuropathic pain. However, typical adverse effects include fatigue, sleepiness, nausea, indigestion, and constipation [11].

Based on the literature review, recent reports are focused on the personalized medicine strategy for CPM. Systematic screening is the first step in personalized CPM, which is followed by an extensive pain evaluation. The etiology and mechanism of pain are carefully characterized in order to inform therapeutic decisions. A tailored response criterion is provided by the documentation of an individual pain target. Furthermore, we can increase adherence and symptom management through long-term monitoring that is customized to the requirements of an individual [12]. Systematic reviews would be beneficial to better investigate how various cancer pain assessment components might enhance treatment results. Understanding the molecular basis of the pain mechanisms and the pain expression pathways will enable us to improve our therapeutic options through pharmacogenomics-based evidence and suggestions. The studied genetic markers have been identified in drug transporters, drug-metabolizing enzymes, cyclooxygenases, opioid receptors, and genes encoding the components of pathways involved in the perception and processing of nociceptive data, the modulation of the pharmacokinetics, or the pharmacodynamic impacts of analgesics [13,14,15]. The current study aimed to review the reports of CPM and shed light on the potential of personalized medicine in cancer treatment by suggesting pharmacogenomics-based makers.

## 2. Materials and Methods

### 2.1. Data and Systematic Strategy

**Figure 1:**
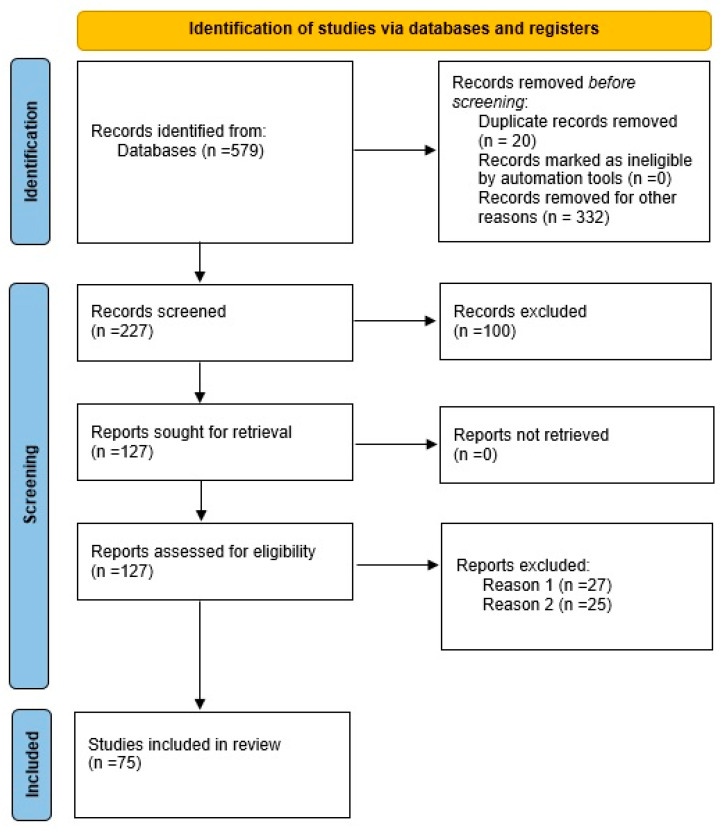
PRISMA flow diagram for systematic reviews. Reasons 1 and 2 were a lack of covering cancer pain management (CPM) and reports with no novel data.

At first, PubMed searched for the keywords “cancer”, “pain”, and “personalized medicine”. The results revealed 597 publications from 1990 to 2023; among them, 40 items were clinical trials, 149 were reviews, and 458 were published in the last 5 years. All 597 publications were studied and categorized into three groups, as follows: (1) papers focused on cancer types, (2) papers concentrated on the types of CPM, and (3) papers including personalized medicine and pharmacogenomics in CPM. The inclusion and exclusion criteria of the investigated papers are summarized in a PRISMA flow chart. The exclusion criteria were duplicated reports, unrelated reports about cancer and pain, papers with pain as one of the side effects or disease manifestations, and some papers that were written in a non-English language, like Chinese or French (Figure 1).

### 2.2. Bioinformatics Analyses

After the literature review and data mining, the current study designed a bioinformatics-based investigation through a step-by-step procedure. At first, the basis of the in silico investigations were built on the two major classifications, including genes involved in the pain pathways and cancer pathways. The cutoff was considered for the top 100 genes extracted from GeneCards (http://www.genecards.org/ (accessed on 20 May 2023)). These 100 genes were prioritized based on the relevance score, which was taken from Elasticsearch 7.11. In the next step, overlapping genes were kept and the others were discarded. The overlapping genes were then tested for their molecular relationships by a string model. The final genes which were in a distinct network utilized as the main gain for further investigations. To cover all possible makers in the personalized medicine perspective of CPM, the association of the aforementioned genes with miRNAs were studied by miRTargetLink 2 (https://ccb-compute.cs.uni-saarland.de/mirtargetlink2/ (accessed on 20 May 2023)). Notably, the final results from miRTargetLink 2 were obtained by setting only strong evidence, and the resulting concentric model was used. In the final step, the overlapping genes that showed reliable relationships and strong evidence from the gene-miRNA concentric model were selected for finding the putative important variants. These variants were searched in PharmGKB (https://www.pharmgkb.org/ (accessed on 20 May 2023)) to find potential plausible variants with pharmacogenomics impacts on CPM.

## 3. Results

### 3.1. Literature Review

The current study systematically reviewed the literature with the keywords cancer, pain, and personalized medicine. After filtering 579 publications indexed in PubMed, 127 related publications remained. In the next step, the papers were categorized into three subgroups, including cancer types in CPM, cancer pain strategies, and personalized medicine in CPM. All three categories are discussed in detail in the following, based on the papers’ findings. Moreover, a bioinformatics-based approach was carried out on the final findings from the review literature and new suggestions for the pharmacogenomic management of CPM. There are abundant studies that mention pain signs in cancer-managing strategies, but studies focused on the introduction of pain in various cancer types were selected and are categorized here. These studies (11 papers) were categorized based on their publication priorities from 2015 to 2022. The types of cancer in these publications were breast cancer (BC), colorectal cancer, pancreas cancer, gastrointestinal cancer, cervical cancer, and bone cancer. Among them, breast and pancreas cancers were the first and the second most repetitive cancers in pain management, respectively. Reviewing 32 papers that deeply studied CPM from 2003 to 2023 revealed that there are three types of strategies conducted for CPM. These strategies can be divided into pharmacological, non-pharmacological, and the combination of pharmacological and non-pharmacological scenarios. Notably, the majority of the reports indicate that personalized care therapy are at the top of the list of strategies. Numerous studies have investigated multiple questionnaire-based assessments and standard guidelines that are specifically concentrated on the categorization of duration, intensity, and interferences of pain. The results of these studies ultimately recommend therapeutic management of cancer-related pain by pharmacological, psychosocial, and physiological methods. Among these studies, palliative care, addiction, drug dosage, and opioid consumptions were the main topics. Searching for personalized care in cancer management led to 32 related papers from 2011 to 2023. Interestingly, 16 papers were from 2020 to 2023, which can be a significant sign for the increasing trend of personalized medicine as a leading strategy for CPM. In this section, the main concentration of related reports is the pharmacogenomics contents and reviewing and introducing the molecular-based suggestions for CPM. The results show that there are 76 genes and 121 variants reported in the literature on CPM as of now. The most repeated genes are CYP2D6, COMT, and OPRM1. The other genes with more than one involved variant in CPM are as follows: ABCB1, ABCC4/MPR4, ANGPT1, AQP7, CACNG2, CYP17A1, FAAH, IL-10, IL-6, KCNK9, NFKBIA, P2RY12, SPON1, and TNF. Notably, some studies have reported the expression impacts of some genes (IL-6 and TNF), lncRNAs (UCA1), and gene products (F13B) as the biomarkers of CPM detection. Additionally, to complete the list of reported genes, this study utilized bioinformatical approaches and databases to introduce new related genes. By combining the bioinformatics results as the suggested in silico findings (level 1) and the literature reports as the experimentally evidenced (level 2), a new potential gene list is introduced (level 3) (Table 1) [16,17,18,19,20,21,22,23,24,25,26].

### 3.2. Bioinformatics Analyses

**Figure 2:**
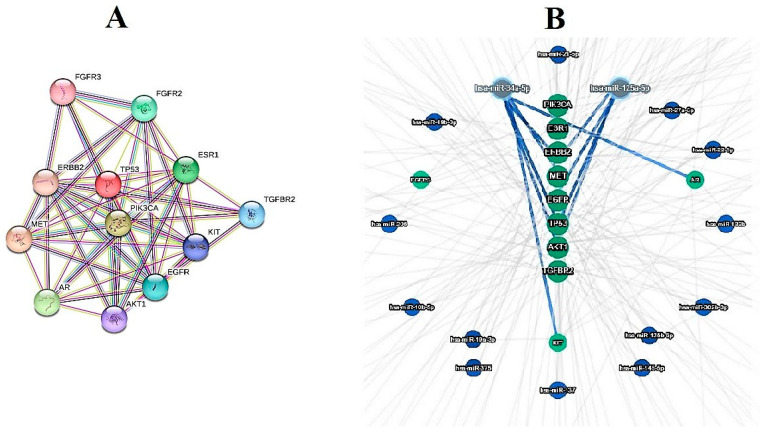
Networks of string model (**A**) and concentric model by miRTargetLink2 (**B**) of first-level genes common in the pain and cancer reports obtained from GeneCards.

**Figure 3:**
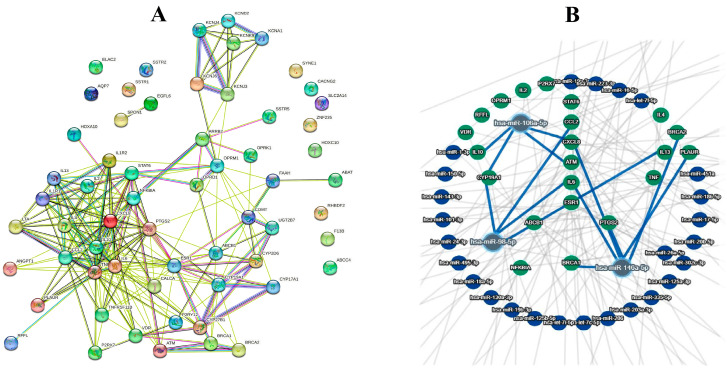
Networks found in the second-level analyses of 46 genes involved in cancer pain management (CPM) extracted from the review literature, represented as a string model (**A**) and concentric model (**B**).

**Figure 4:**
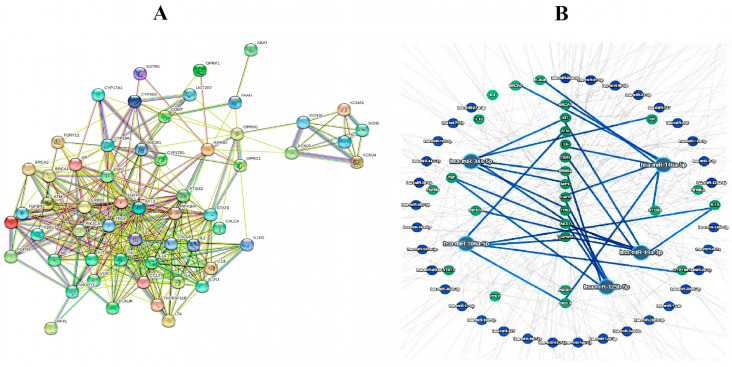
In silico findings of third-level investigation resulting from the combination of first and second levels, displayed in a string model, including 57 completely connected genes (**A**), and gene–miRNA interactions in a concentric model (**B**).

In silico analyses were carried out at all three levels by a string model and miRTargetLink2. At the first level, the data mined from the databases were analyzed. At the second level, the data gathered from the CPM literature review were analyzed. At the final level, the raw data of the first and second levels were merged together and then were analyzed in a unique algorithm of analysis. As mentioned before in Section 2, the results of the first-level analysis were as follows: 12 overlapped genes were found, including ESR1, EGFR, AR, ERBB2, TGFBR2, TP53, FGFR2, MET, FGFR3, KIT, PIK3CA, and AKT1. Additionally, the gene miRNA investigation represented two miRNAs with a high connection level, including hsa-miR-34a-5p and has-miR-125a-5p (Figure 2). The second level analyses indicated that among the 76 genes obtained from the literature review, 46 genes were connected in a united network of the string model. miRTargetLink 2 assessments, based on strong evidence, showed hsa-miR-146a-5p, hsa-miR-106a-5p, and has-miR-98-5p as the most related miRNAs in the concentric model, resulting from 46 aforementioned genes (Figure 3). In the last level of computational predictions, 12 genes from level 1 and 46 genes from level 2 were combined with each other and the string model showed that, surprisingly, all of the combined genes (57 genes without duplications) were related in a unique network of the string model. Furthermore, the miRTargetLink 2 output indicated that there are potential miRNAs linked with more than three targets, such as has-miR34a-5p, hsa-miR-146a-5p, hsa-miR-106a-5p, hsa-miR-125b-5p, and hsa-mIR-19a-3p (Figure 4). It can be concluded that the most important miRNAs in the CPM are has-miR34a-5p, and hsa-miR-146a-5p, which were repeated at all three levels of the current in silico prediction.

As a complementary pharmacogenomics-based analysis, the current review investigated all variant annotations of the final aforementioned 57 genes in PharmGKB. Multiple cutoffs were considered for the variant filtering, including their significance status, association with pain, and a *p*-value lower than 0.05. Finally, 4640 variant annotations were obtained from the PharmGKB database, and 1874 variant annotations were significant. Among these annotations, 68 annotations had an association with pain. After deleting the duplicated variant annotations and performing the cutoffs, 21 variants remained. All of these variants were checked with the reports of the previous data, which are discussed in the present study, and 9 variants remained and were finally added to the previously found variants involved in the CPM (Table 2).

## 4. Discussion

This review emphasizes the importance of personalized medicine in CPM. Also, the findings indicate that the 57 resulting genes from combining the first and second levels of the in silico investigations can be considered the primary gene list for the NGS analysis of pharmacogenomics-based analysis of cancer patients using a CPM strategy. These genes are as follows: ANGPT1, ATM, CALCA, CCL2, CXCL8, CYP27B1, ESR1, IL10, IL13, IL1R1, IL1R2, IL2, IL4, IL6, LTA, NFKBIA, P2RX7, P2RY12, PLAUR, PTGS2, RFFL, STAT6, TNF, TNFRSF11B, VDR, ARRB2, KCNA1, KCND2, KCNJ3, KCNJ4, KCNJ6, KCNK9, SSTR5, ABAT, ABCB1, BRCA1, BRCA2, COMT, CYP17A1, CYP19A1, CYP2D6, FAAH, OPRD1, OPRK1, OPRM1, UGT2B7, EGFR, AR, ERBB2, TGFBR2, TP53, FGFR2, MET, FGFR3, KIT, PIK3CA, and AKT1. Additionally, among the 127 variants that were found by both the literature review and data mining in the PharmGKB database, 21 variants were missense, frameshift, or regulatory variants. These variants are more important than the others in the non-coding sites (introns, 5′UTR, upstreams, 3′UTR, downstreams, and intergenic) at least for whole-exome sequencing (WES) tests. These variants include the following genes; ABCB1 (rs1045642 and rs2032582), AQP7 (rs76608797), COMT (rs4680; Val158Met), CYP2D6 (rs35742686, *2, *3, *4, *5, *6, *7, *8, *9, *10, *11, *15, *17, *29, and *35), FAAH (rs324420 and rs4660928), OPRM1 (rs79910351; Arg181Cys and rs1799971; Asn40Asp), PLAUR (rs4760), and UGT2B7 (rs7439366). Notably, as mentioned before, considering the cancer types in CPM will be beneficial for various aspects. The benefits will be as follows: some cancer types have been investigated very much and some have been neglected; some cancer types with more documentation need more molecular-based approaches in detection and treatment; primary gene lists (gene panels) can be designed for a specific type of cancer from our results on CPM; finally, working groups such as the American College of Medical Genetics (ACMG) and the Clinical Pharmacogenetics Implementation Consortium (CPIC) can categorize their recommendations for a specific cancer type in CPM based on the collected data in this study.

An international classification system for cancer pain was established to better assess cancer pain through the identification of the characteristics of patients and pain syndromes linked to the complexity of CPM. This system later underwent numerous updates. According to the five factors included in the most recent classification system (pain mechanisms, incidental pains, psychological distresses, addictive behaviors, and cognitive functions), patients are classified as having a suitable, acceptable, or weak prognosis for managing pain, according to the interactions of these factors [48,49,50,51,52]. Numerous challenges are relevant to assessing pain in cancer patients. Treatments should be examined for potential medication interactions and adverse effects, specifically if opioids are being added for pain treatment. Many characteristics, such as breakthrough pain, neuropathic pain, addiction background, psychological distress, tolerance, and the occurrence of delirium, have been suggested as predictive for pain control [53]. Once pain management is implemented, it is essential to review the patient’s pain and results (pain alleviation, adverse effects, physical and psychosocial actions) on a regular basis. This is regarded as the most essential part of pain management. Opioids and other medications are titrated based on these assessments to maintain a desirable balance of effectiveness and adverse effects [54,55]. The present study systematically reviewed the literature about CPM and divided the studies into three categories, which are discussed here. As mentioned before, 11, 32, and 32 publications according to the cancer types, CPM strategies, and personalized medicine in CPM, respectively, were found to be related to CPM.

### 4.1. Cancer Pain Management Strategies

Cepeda et al. investigated if there was any correlation between the calculated percentage of pain reduction (CPPR) and the patient-reported percentage of pain reduction (PRPPR). Patients with acute or cancer pain were requested to rank the severity of their pain on a 0–10 verbal numerical rating scale (NRS) and to indicate the percentage of pain decrease from the initial pain following analgesic administration [56]. Musshoff et al. showed that hair analysis could serve as a valuable and supplementary method for isolating patients who take opioid analgesics for pain relief. They found fentanyl and tramadol levels in their participants [57]. Dalal and Bruera found that cancer-related pain is a multidimensional construct resulting from a complicated combination of physiological, socio-cultural, psychological, behavioral, sensory, and cognitive factors. Pain management interventions will be most efficient when therapies are personalized to the many physical and non-physical aspects of cancer pain, and the patient/family is educated and involved in the decision-making [58]. Sarzi-Puttini et al. stated that selecting an effective, suitable, personalized analgesic prescription for individuals with chronic pain is feasible and will improve compliance, general functioning, and QoL [59]. According to Westerling, multidisciplinary rehabilitation and personalized pain management may enhance cancer survivors’ QoL [60]. Petersen et al. found 337 pain items in the literature, as well as 29 new questions suitable for the quality of life questionnaire (QLQ)-C30. They prepared an item bank of 16 pain-measurement items appropriate for computerized adaptive testing (CAT), such as, “Have you had any trouble falling asleep because of pain?” The novel item bank will greatly increase pain assessment precision while being backward compatible with the QLQ-C30 questionnaire. They proposed starting CAT measurements with a pain test utilizing the two original QLQ-C30 pain items (pain interference and pain intensity). The pain CAT from the European Organization for Research and Treatment of Cancer (EORTC) is now available for “experimental” uses [61]. Balducci and Dolan investigated palliative care for disease in elderly patients and concluded that the purpose of palliative care is healing, which may be attained even when cancer is incurable. As cancer-related mortality and treatment problems grow with age, palliative care becomes increasingly important in the care of elderly cancer patients. Target planning, symptom control, and caregiver attention are the three foundations of effective palliative care [62]. According to Bhatnagar and Gupta, integrating cancer pain and symptom management into present pain management, fellowships, and introducing a comprehensive pain and palliative care paradigm at all levels of the healthcare system are priorities. Simultaneously, it is critical to conduct research, collect information, and develop guidelines and suggestions for accurate symptom management across a wide range of patients and diseases in order to provide a personalized strategy for patient care [63]. Arthur et al. worked on an investigation of the relationship between the Edmonton Classification System for Cancer Pain (ECS-CP) characteristics and pain treatment results among outpatients. It is noteworthy that research on the ECS-CP has demonstrated that it may predict the complexity of pain management using five characteristics: pain mechanism, psychological distress, incident pain, addictive behavior, and cognitive function. They discovered that neuropathy was a poor predictive factor in the treatment of advanced cancer pain. Furthermore, once the pain was treated by a palliative care expert, the sum of ECS-CP characteristics did not predict pain management at the follow-up appointment, despite being related to increased opioid and adjuvant analgesic usage at referral [64]. According to Liu et al., a realistic, personalized management strategy may then be constructed to treat the pain with the proper analgesics, with objectives for therapy established. They emphasized that, while many treatment options are available, care strategies must be tailored to each individual patient. Few acceptable studies have reviewed the current cancer pain treatments, which is likely due to the difficulties of performing sufficiently powered randomized controlled trials for the different patient ethnicities [65]. Colvin underlined that although potential emerging drugs, including histone deacetylase 6 (HDAC6) inhibitors, are now in early-phase clinical studies for cancer therapy, no preventative treatments have demonstrated meaningful clinical benefit. Repurposing drugs such as metformin may provide an additional treatment route. There are few effective treatments available for painful chemotherapy-induced peripheral neuropathy (CIPN). The American Society of Clinical Oncology has lately recommended duloxetine. Particularly, during oncological treatment, any new therapies adopted must not conflict with the tumoricidal impacts of chemotherapy. In comparison to other neuropathic pain types, this presents a further hurdle [66]. Vimalnath et al. described the production and investigation of Ce-141 as an effective theragnostic agent for metastatic skeletal lesions. They proved the potential value of 141Ce-DOTMP as a theragnostic component for the tailored patient treatment of cancer patients with painful metastatic skeletal lesions [67]. Sica et al. studied the efficiency of the intrathecal pump in 140 patients undergoing pain management. They found that intrathecal therapy is one of the most effective options for managing and treating severe chronic refractory pain. Overall, this therapy is safer than systemic opioids, which frequently require greater dosages to be efficient, leading to probable major side effects [68]. Interestingly, Miller and her colleagues claimed that acupuncture reduced pain and other side effects associated with cancer. Those who had advanced diseases and greater initial pain levels were more probable to experience considerable pain relief [69]. Cuomo et al. suggested a novel model called the “trolley analgesic model”, which permits the employment of personalized therapies with dynamic multimodal methods for pain management based on (1) the severity of pain, (2) the physiopathology of pain, (3) the complexity of symptoms, (4) the existence of comorbidities, and (5) physiopathological factors and social conditions [70]. Vitzthum et al. showed the ability to predict negative opioid-related results in cancer survivors. Personalized risk-stratification techniques, with additional verification, could direct treatment when prescribing opioids for cancer patients [71]. According to LeBaron et al., Behavioral and Environmental Sensing and Intervention for Cancer (BESI-C) has the ability to track and predict pain while also improving self-efficacy, safety, communication, and QoL in cancer patients [72]. Chapman and Beach emphasized the need for combining communicative pain research with ongoing attempts to improve more personalized treatment approaches [73]. According to Oldenmenger et al., the Breakthrough Pain Assessment Tool (BAT) is a legitimate and precise questionnaire that may be used in everyday practice to measure breakthrough pain in Dutch cancer patients. They examined nine BAT questions, such as “How often do you get breakthrough pain?” and “How long does a typical episode of breakthrough pain last?” [74]. O’Connor et al. thought that if a tailored pain target is included in the CPM plan, healthcare practitioners may accommodate the assumption that patients will self-report the pain [75]. Ben-Arye et al. investigated the outcomes of an integrative oncology (IO) therapy program that was personalized and offered to 815 eligible patients receiving cancer treatment in adjuvant, neo-adjuvant, and palliative care settings. They observed that after an initial consultation with an integrative clinician and follow-up visits, a personalized integrative oncology program with high compliance may lead to good pain relief after 6 weeks, with none to minimal benefit after 12 weeks. Patients receiving adjuvant and neo-adjuvant chemotherapy in addition to patients receiving palliative care all benefited more from the treatment at 6 weeks in the high-adherence group [76]. A complicated interplay among biological reasons, neurological alterations, and environmental influences can result in pain, according to Tang and Tanco after analyzing the effects of addiction and individualized care in the treatment of CPM [10]. Mao et al. investigated the efficacy of electroacupuncture or auricular acupuncture against standard care in the treatment of chronic musculoskeletal pain in cancer survivors. Electroacupuncture and auricular acupuncture reduced pain more effectively than standard therapy in this randomized clinical study of cancer survivors with persistent musculoskeletal pain. Auricular acupuncture, on the other hand, did not show superiority to electroacupuncture, and patients who had it experienced greater side effects [77]. Soto-Perez-de-Celis et al. showed that a patient navigator (PN)-led multifunctional treatment significantly enhanced the availability of supportive and palliative treatment among Mexican patients with metastatic solid tumors when compared to standard oncological care alone. Furthermore, the PN-led intervention boosted AD completion while decreasing the number of patients experiencing moderate or severe pain. At 12 weeks, the therapy did not substantially enhance QoL when compared to standard oncological treatment alone [78]. Liu et al. investigated the practice, knowledge, and attitudes of healthcare providers (HCPs) toward pharmacists and advanced methods of CPM and found that HCPs’ levels of practice, information, and attitudes regarding pharmacists and advanced techniques of CPM were average in China; however, pharmacists had the worst performance, indicating a need for further enhancement [79]. Similar to Liu et al.’s study, Xie et al. investigated CPM among healthcare workers, including physicians, pharmacists, and nurses in China, and found that multidisciplinary teamwork and the use of mobile devices can help to advance and improve CPM [80]. Batistaki et al. investigated the relationships among breakthrough cancer pain (BTcP), background cancer pain, and analgesic therapy. They emphasized how pain exacerbations should be properly observed, and how to distinguish BTcP from changes happening during opioid titration, end-of-dose failure, and circadian fluctuations. They noted that, despite the fact that various recommendations and guidelines on the nomenclature, diagnosis, and management of BTcP have arisen over the last decade, there are still many concerns to be addressed. Early detection, thorough monitoring of pain intensity and etiopathogenetic features, as well as accurate evaluation of the forms of pain, are all essential. To attain optimal pain management and an improved QoL for cancer patients, they recommended a multimodal analgesic approach [81]. Masukawa and colleagues established machine learning models for CPM and discovered that the models could predict social pain, spiritual pain, and severe signs in terminally suffering cancer patients using text data from electronic healthcare records [82]. In addition to the previously stated studies on CPM with acupuncture by Miller et al. and Mao et al., Yang et al. performed a study on the efficacy of acupuncture versus standard treatment on the quality of sleep in cancer survivors with chronic pain. They discovered that electroacupuncture and auricular acupuncture caused a clinically meaningful and long-lasting enhancement of sleep quality in cancer survivors with concomitant sleep disruption and chronic pain. Acupuncture, according to their findings, may be an evidence-based nonpharmacologic strategy for improving sleep health in cancer survivors who are in pain [83]. The preliminary findings from a remarkable trial by Reddy et al. indicated that cancer patients might effectively transition from opioids to levorphanol utilizing an opioid rotation ratio (ORR) of 8.5. Levorphanol was well tolerated and linked to better pain and symptom management [84]. Aziz and Cascella’s study on peripheral neurolytic blocks indicated that some forms of painful diseases, such as pancreatic neoplasia pain, must inevitably be handled through the administration of less invasive analgesic procedures, even before symptoms appear. Neurolysis is now defined as the targeted, iatrogenic destruction of brain tissue to provide pain alleviation. Actually, the understanding of nerve pathology and the development of methods and tools that are accessible have inundated the indications for these techniques throughout time. For instance, improvements in medical imaging have made interventional pain management more precise and, thus, more effective. Peripheral neural blockade and neuro-destructive methods have been gradually included in early pain management algorithms. Once more, peripheral nerve blocking is a treatment option for treating the spasticity of different muscles [85]. According to Dalal et al., the majority of cancer patients who experienced pain were able to express their ideal level of pain reduction on a scale from 0 to 10. Over the course of our follow-up period, the median personalized pain goal (PPG) remained at 3 and was quite consistent. The PPG represents a new target for pain experience. A regular PPG recording may help with personalized pain management [86]. A brief description of the most important studies in this category is summarized in Table 3.

### 4.2. Personalized Medicine in Cancer-Pain Management

Ling and Larssen evaluated 82 patients suffering from head and neck cancers who took radiation for pain caused by oral mucositis (OM). Stepwise combinations of acetaminophen, nonsteroidal anti-inflammatory drugs (NSAID), and opioids were used. According to the questionnaire responses, personalized pain therapy with systemic analgesics applied to the greatest degree possible was inadequate [87]. Khan et al. compared opioids with adjuvant analgesics (AA). They concluded that AA is an important strategy in CPM. AAs may be used alone in some cases, such as peripheral neuropathies; however, when administered separately for cancer pain, AAs are seldom sufficient analgesics, and a typical medical procedure requires the administration of both an opioid and an adjuvant. AAs increase the therapeutic ratio of opioids in this environment by improving analgesia and decreasing adverse effects. Their primary responsibility is the long-term care of cancer pain syndromes. AAs, despite opioids, can cause substantial end-organ damage. They function in ways that are unique from opioids and from one another. AAs are present at every level of the WHO three-step ladder, and effective CPM depends on their effective administration [88]. Galvan et al. examined the concept that genetic variants might regulate individual responsiveness to opioid medications among 1,008 cancer patients. Utilizing an SNP-array, they examined 1 million single-nucleotide polymorphisms (SNP) in European cancer patients. Association analysis indicated that eight SNPs significantly were associated with pain decrease, including rs13421094, rs12211463, rs7757130, rs2473967, rs2884129, rs7104613 (SPON1 gene), rs12948783 (RHBDF2), and rs10413396 (ZNF235). Among them, rs12948783 (the upstream of the RHBDF2 gene) represented the best statistical association. Their findings suggested that the found SNP panel can alter how cancer patients respond to opioid therapy and may offer a new method for treating cancer pain on a personalized strategy [45]. Heintzlman et al. reported that longitudinal analysis of pain in patients with metastatic prostate cancer employing natural language processing of medical record text has significantly improved the detection and comprehension of disease phenotypes and their association with genetic and non-genetic factors. Their research demonstrated the viability and generalizability of natural language processing (NLP)-based monitoring of patients’ pain state, and it offers some phenotype-oriented insights helpful for directing future research [89]. Hui and Bruera stated in a review that CPM starts with systematic screening, followed by a thorough pain evaluation. They stated that adherence and symptom management can be increased by using longitudinal monitoring that is personalized to the requirements of the individual. The relevance of electronic diaries for pain evaluation and pain clinical pathways, they continued, has to be thoroughly examined. The pain expression pathway was discussed, which explains how pain caused by tissue damage is transferred via afferent pathways, recognized in the somatosensory cortex, and then expressed by the patient. Clinically, pain expression is the only result that can be molecularly evaluated [12]. According to Skorpen et al., anesthesiologists and pain specialists should be informed of the Arg181Cys mutation in the opioid receptor (MOR) as a potential cause of opioid ineffectiveness and should take genotyping into consideration in cases where it is appropriate, such as when patients report a family history of inadequate opioid pain relief [42]. In their review of the function of cytochrome P450 pharmacogenomics in patients with persistent non-cancer pain, Tverdohleb et al. placed special emphasis on the genotyping of CYP2D6 expression and how its high polymorphism affects how opioid drugs are metabolized [90]. Sivanesan and Gitlin, in their review, aimed to elevate the understanding of desmoid tumors and debated pharmacotherapeutic administration. They highlighted the evidence of Wnt signaling pathway involvement, APC, and β-catenin in desmoid tumors. They underlined tramadol and ziconotide as the primary analgesic suggestions for pain alleviation, highlighting the opioid receptor mu-1 (OPRM1) as the major target of the analgesics [91]. With the exception of codeine and tramadol, Obeng et al. analyzed the pharmacogenomics-based publications on CPM and proposed that, in decreasing order, the examples of morphine-OPRM1, oxycodone-CYP2D6, and hydrocodone-CYP2D6 might be the ones that are closest to clinical application. Additional studies on hydrocodone are recommended, according to their findings, as it is one of the opioids in pharmacogenetics that has received the least attention [92]. Haji et al. found an association between the c.118A>G variant (OPRM1) and the course of morphine treatment, morphine dosages, and QoL in palliative cancer pain conditions. They found that neither the morphine dosages nor the duration of morphine treatment changed substantially between cancer types or individuals with the AG genotype c.118A>G. OPRM1 individuals required a larger morphine dosage than AA patients. Furthermore, metastases, the OPRM1 SNP, age, and sex were all related to QoL [43]. Mosley et al. utilized the capabilities of the CYP2D6*2, *3, *4, *6, *7, *8, *9, *10, *11, *15, *17, *29, *35, *41 alleles, *5 (gene deletion), and gene duplication in their genotyping platform. They also validated that CYP2D6 variants affected the CPM [37]. Yang et al. reviewed the data on the association of genetic variants with cancer pain until 2019. In their valuable paper, they reported the genetic variants associated with different pain-causing cancers, which is summarized in Table 2 [30]. Nissenbaum et al. claimed that certain polymorphisms in the CACNG2 gene are linked to the likelihood of experiencing prolonged postmastectomy pain (PMP) following breast surgery [93]. Bortsov et al. concluded that the A-C-C haplotype of three variants in the CACNG2 gene (rs4820242, rs2284015, and rs2284017) is associated with an elevated chance of developing PMP [32]. The study by Lee et al. focused on finding vulnerable loci and enriched pathways for clinically relevant acute post-adjuvant radiotherapy (RT) pain, characterized by moderate to severe pain (pain score ≥ 4) at the end of RT. In 1112 breast cancer patients, they performed a genome-wide association study (GWAS) using 1,344,832 SNPs. Their findings showed that four SNPs were suggestively associated with post-RT pain; rs16970540 in RFFL or near the LIG3 gene, rs4584690, and rs7335912 in the ABCC4/MPR4 gene, and rs73633565 in the EGFL6 gene. The genomics investigation suggested that neurotransmitters, cytochrome P450, and olfactory receptors could be involved in post-RT pain, but functional evaluation revealed that glucuronidation and olfactory receptor activity were the most substantially enriched biological characteristics [28]. Genovese and Mao demonstrated that specific genetic variants, including rs4680 (missense of COMT gene) and rs2369049 (intergenic) were correlated with response to acupuncture-type intervention for arthralgia management [47]. De Santos et al. investigated the efficacy of the immediate-release fixed combination of oxycodone/acetaminophen (OxyIR/Par) for the treatment of moderate-to-severe intensity background pain in cancer patients with BTcP when used either by itself or in combination with other strong opioids. They discovered that a low dose of the fixed combination of OxyIR/Par was efficient alone or in conjunction with other opioids [94]. In a remarkable report, Xu et al. 2020 identified the herbal groups active in pain disorder subtypes through machine learning, which uncovered new molecular mechanisms of algesia. They performed multiple investigations to show that the overlap and interaction of herb-related targets (Htargets) and opioid targets (Otargets) were promising for treating pain without herb resistance. Indeed, six Otargets were found in a module, including OPRM1, OPRK1, OPRD1, SSTR1, SSTR2, and SSTR5, which connected to 33 Htargets (including CXCL10, CCR3, CCR5, NPY, PDYN, SLC6A3, DRD2, HTR1A, etc.). GO analysis revealed that the Htargets enriched three more pain-related pathways compared to the Otargets, including neuroactive ligand–receptor interaction, the cAMP signaling pathway, and the sphingolipid signaling pathway, demonstrating that pain-related herbs have the ability to overcome opioid tolerance [95]. Hasuo et al. studied alexithymia, which is having trouble recognizing and describing feelings and sensations, contributes to an increased risk of chronic pain. They observed that 36.2% of participants had alexithymia. Based on this finding, it can be concluded that there are serious challenges in reflecting some individuals’ pain. This can be a clue for the weakness of pain questionaries, and it highlights the importance of the personalized-medicine based treatment of patients with cancer pain [96]. De Bono et al. investigated males with metastatic castration-resistant prostate cancer who were taking a novel hormonal treatment (such as enzalutamide or abiraterone) at the time of disease development. They discovered a delay in pain progress in the Olaparib group, representing a direct patient advantage for Olaparib (PARP inhibitor) against the control medication in patients with at least one BRCA1, BRCA2, or ATM mutation [97]. Bugada et al. reviewed the literature and summarized the available evidence on genetic variations and opioid response by the year 2020. They concentrated on the pharmacogenetic framework and its therapeutic implications, emphasizing how it could lead to more appropriate opioid prescription in cancer patients. They discovered that OPRM1 and COMT gene variants influenced both pain perception and opioid responsiveness. Cancer patients who had at least one A allele (OPRM-A118G) and a Met allele (COMT-Val158Met) appeared to have less pain and analgesia, as well as fewer side effects. Other genes associated with drug transport and metabolism, either alone or in combination, were likely to have an influence on the clinical outcome [33]. Rienzo et al. investigated the possible relevance of PRDM12 in CPM due to its simultaneous participation in both nociceptive and cancer-developing pathways. Pharmacotherapies targeting PRDM12-involved pathways or the epigenetic modifications regulated by PRDM12 might be a potential technique in the treatment of chronic pain syndromes [98]. From 2012 to 2018, Reizine et al. examined 61,572 adult cancer patients for opioid consumption. They verified the findings of Soley et al. on the pharmacogenomics effects of CYP2D6 in CPM and indicated that CYP2D6 genotype may identify cancer patients at higher risk of insufficient analgesia when treated with standard first-line opioids, such as codeine, tramadol, or standard dose hydrocodone [99]. In 174 advanced cancer patients receiving supportive treatment, Yennurajalingam et al. examined the genetic characteristics associated with pain intensity, necessary daily opioid dosage, and pain response. The daily intake of opioids was correlated with the variants summarized in Table 2 [31]. To find out if putative pain biomarkers might be found in patient serum and whether they are associated with certain pain patterns, Saloman et al. designed an investigation. Hierarchical cluster analysis showed a subset of patients with mainly constant, mild to moderate pain, indicating increased interleukin-1β (IL1β), interleukin-6 (IL6), interleukin-2 (IL2), tumor necrosis factor alpha (TNF), and monocyte chemoattractant protein-1 (MCP1), whereas patients with higher interleukin-4 (IL-4), interleukin-8 (IL-8), and calcitonin gene-related peptide (CGRP) were more likely to exhibit severe pain. Surprisingly, the assessments of each individual biomarker showed that fractalkine and TNF levels in the blood were lower in individuals with chronic pain. TNF was much lower in patients with severe pain, while IL-6 and substance P levels trended downward [100]. In their observational study, Crescioli et al. found that the SNPs of IL6 (rs1800797) and TNF (rs1800629) may serve as possible indicators of baseline pain severity and opioid dosage requirements among pediatric cancer patients [40]. In a pioneer study, Chang et al. investigated the putative participation of cancer-related lncRNAs in endometriosis and detected genetic variants in UCA1, a lncRNA serving as a miRNA sponge, which can be found in endometriosis development and is potentially associated with infertility via regulating lipogenesis [101]. Satkunananthan et al. conducted a review to assess the polymorphism associations with variability in opioid therapy responses in Asian cancer pain patients. Their findings indicate that CYP2D6 *10 had the most therapeutic impact in tramadol care. They recommended that OPRM1 (rs1799971), COMT (rs4680), and ABCB1 (rs1045642, rs1128503, and rs2032582) should be investigated deeper for their importance in Asian populations in future studies [27]. There are 38 single nucleotide polymorphisms (SNPs) in the ABCB1 coding region; the three most prevalent and well-studied variants to date are C3435T on exon 26 (rs1045642), G2677T on exon 21 (rs2032582), and C1236T on exon 12 (rs1128503), with varying allelic frequencies in various populations [102,103]. Allelic P-gp variations are also correlated with changed P-gp expression at the blood brain barrier (BBB), influencing drug delivery to the central nerve system (CNS), as is the case with opioids, leading to inter-individual diversity in pain relief [104,105]. Carriers of the 1236TT, 2677TT, and 3435TT SNPs (also known as the “TT-TT-TT” haplotype) require greater methadone doses to prevent withdrawal, which is likely due to a faster metabolism and less methadone plasma levels; heterozygous individuals for these three SNPs have about a three-fold chance of sustaining a lower methadone dose. SNP 1236C > T is a synonymous variation found in one of the intracellular loops of the protein, near an ATP-binding/utilization site. Although 1236C > T does not modify the protein sequence, it might have an influence on P-gp translation, regulation, and the stability of RNA [102,103]. Homozygous C3435T TT carriers, on the contrary, exhibited superior analgesic impacts with morphine administration than wild-type CC individuals, although they were reported to have a higher incidence of chronic postoperative pain [106,107]. In 2020, CPIC released a guideline for CYP2D6, OPRM1, and COMT genotypes and selecting opioid treatment, which provided therapeutic guidelines for the utilization of CYP2D6 genotype outcomes during prescribing codeine and tramadol. They described the limited and/or inadequate data for CYP2D6 and hydrocodone, oxycodone, and methadone, and also for OPRM1 and COMT in clinical routine [108].

Wang and his colleagues found that oral oxycodone can produce unusual alterations in cytokine levels and gut microbiota in individuals with medium moderate to severe cancer pain, leading to persistent systemic inflammation. Continuously oxycodone usage may result in analgesic resistance due to the persistent elevation of IL-6 and TNF-α levels [109]. In a GWAS, Nishizawa et al. revealed SNPs and a gene related to opioid analgesic requirements for the CPM, including the ANGPT1 SNPs (rs1283671 and rs1283720) and the SLC2A14 gene [110]. Tang et al. identified macrophage-specific Smad3 as a crucial regulator for promoting the macrophage to neuron-like cell transition (MNT) at the genomic level using single-cell RNA sequencing; its disruption successfully inhibited the tumor innervation and cancer-dependent nocifensive actions in vivo. They suggested that MNT may serve as a precisely targeted therapy approach for cancer pain [29]. Rahmioglu et al. performed a GWAS meta-analysis, which included 60,674 cases and 701,926 controls of European and East Asian ethnicity, and found 42 genome-wide significant loci with 49 unique associated signals [38]. In 80 male patients with severe postoperative sufentanil use or acute pain, Li et al. used label-free proteomics to identify 29 distinct preoperative serum proteins. They identified a number of distinct proteins that were connected to postoperative acute pain and were active in inflammatory pathways, blood coagulation cascades, and extracellular matrix (ECM)-related activities. They also noted that F13B could be a novel marker for acute postoperative pain [111]. Similar to the previous section, these data from the most important studies are summarized (Table 4).

In summary, the effects of variations on CPM can be categorized in some main topics, including neural conduction, the effects of various medications, and metabolism processes. Notably, there are some limitations CPM faces that should be considered in future studies, including an evaluation of the differences between genotype and phenotype, the economic costs of the number of determinations presented and the cost–benefit balance as a possible limitation of the implementation, the relative frequency of variability points and their contribution to therapeutic improvement as a possible additional point to investigate and compare, and temporary delays in performing some determinations compared to the rapid evaluation necessary in pain management. Additionally, monitoring and measuring pain remains a noticeable concern that needs more accurate procedures than those described here, such as various questionnaires. An important factor in genotyping cancer pain patients is considering the population stratifications, which means variabilities in the allele frequencies of the potential variants suggested for CPM in different ethnicities.

## 5. Conclusions

The current systematic review investigated 75 publications in 3 categories related to CPM. This review highlights the trend of development and tendency of the literature on personalized medicine aspects in CPM as the main and high-level strategy for CPM, and all of their challenges. Finally, together with the literature reports and in silico assessments, the pharmacogenomics-based aspect of CPM is highlighted in both the prognosis and diagnosis of cancer patients reacting to various kinds of pain. A primary gene list (PGL) consisting of 57 potential genes in CPM was introduced for NGS analysis and narrowed by missense and important coding variants to the 21 variants for the WES test. Complementary computational predictions suggest that hsa-miR-34a-5p and hsa-miR-146a-5p might be considered as the putative circulating biomarkers of CPM, which needs to be more investigated by future studies.

## Figures and Tables

**Table 1:** The genes obtained from data mining and literature review and final combination of the primary gene list for cancer pain management (CPM) strategies.

Level 1	Level 2	Level 3
ESR1	ANGPT1	ANGPT1
EGFR	ATM	ATM
AR	CALCA	CALCA
ERBB2	CCL2	CCL2
TGFBR2	CXCL8	CXCL8
TP53	CYP2D6, CYP19A1, CYP17A1, CYP27B1	ESR1
FGFR2	ESR1	IL10, IL13, IL2, IL4, IL6, IL1R1, IL1R2
MET	IL2, IL4, IL6, IL10, IL13, IL1R1, IL1R2	AKT1
FGFR3	LTA	LTA
KIT	NFKBIA	NFKBIA
PIK3CA	P2RX7	P2RX7
AKT1	P2RY12	P2RY12
	PLAUR	PLAUR
	PTGS2	PTGS2
	RFFL	RFFL
	STAT6	STAT6
	TNF	TNF, TNFRSF11B
	TNFRSF11B	VDR
	VDR	ARRB2
	ARRB2	KCNJ3, KCNJ4, KCNJ6, KCNK9, KCNA1, KCND2
	KCNA1, KCND2, KCNJ3, KCNJ4, KCNJ6, KCNK9	SSTR5
	SSTR5	ABAT
	ABAT	ABCB1
	ABCB1	BRCA1, BRCA2
	BRCA1, BRCA2	COMT
	COMT	CYP2D6, CYP17A1, CYP19A1, CYP27B1
	FAAH	FAAH
	OPRD1, OPRK1, OPRM1	OPRD1, OPRK1, OPRM1
	UGT2B7	UGT2B7
		EGFR, FGFR2, FGFR3
		AR
		ERBB2
		TGFBR2
		TP53
		KIT
		PIK3CA
		MET

Levels 1, 2, and 3 refer to data mining from GeneCards (from bioinformatics), review literature (from previous investigations), and combination of levels 1 and 2, respectively.

**Table 2:** Final gene list and their associated variants in pharmacogenomics of cancer pain management.

Gene	Variant	Function	Author, Year	Country	Reference
ABAT	rs1641025	Intronic	Satkunananthan et al., 2022	Asian	[27]
ABCB1	rs1045642	Missense	Satkunananthan et al., 2022	Asian	[27]
ABCB1	rs1128503	Synonymous	Satkunananthan et al., 2022	Asian	[27]
ABCB1	rs2032582	Missense	Satkunananthan et al., 2022	Asian	[27]
ABCC4/MPR4	rs4584690	Intronic	Lee et al., 2019	European-Americans, Nigeria; Han Chinese; and Japanese	[28]
ABCC4/MPR4	rs7335912	Intergenic	Lee et al., 2019	European-Americans, Nigeria; Han Chinese; and Japanese	[28]
ANGPT1	rs1283671	Upstream	Tang et al., 2022	Cell line	[29]
ANGPT1	rs1283720	Upstream	Tang et al., 2022	Cell line	[29]
AQP7	rs76608797	Missense	Yang et al., 2019	NA	[30]
AQP7	rs33386144	Intergenic	Yang et al., 2019	NA	[30]
ARRB2	rs1045280	Intronic			[31]
ATM	rs11212570	Intronic	Yang et al., 2019	NA	[30]
CACNG2	rs2284017	Upstream			[32]
CACNG2	rs4820242	Upstream	Yang et al., 2019	NA	[30]
CACNG2	rs2284015	Upstream	Yang et al., 2019	NA	[30]
CACNG2	rs2284017	Upstream	Yang et al., 2019	NA	[30]
COMT	rs4680 (Val158Met)	Missense	Yang et al., 2019	NA	[30,33]
COMT	rs165774	Downstream	Yang et al., 2019	NA	[30]
COMT	rs887200	Intronic	Yang et al., 2019	NA	[30]
COMT	rs4818	Synonymous	Yang et al., 2019	NA	[30]
COMT	rs9306234	3’UTR	Yennurajalingam et al., 2021	USA	[31]
COMT	rs165728	3’UTR	Yennurajalingam et al., 2021	USA	[31]
COMT	rs2020917	Upstream	Yennurajalingam et al., 2021	USA	[31]
COMT	rs2075507	Upstream	Rakvåg et al., 2008	Caucasian	[34]
COMT	rs4633	Synonymous	Tchivileva et al., 2011	Caucasian	[35]
COMT	rs6269	5’UTR	Tchivileva et al., 2011	Caucasian	[35]
CXCL8	rs4073	Upstream	Yang et al., 2019	NA	[30]
CYP17A1	rs4919686	Intronic	Yang et al., 2019	NA	[30]
CYP17A1	rs4919683	Intronic	Yang et al., 2019	NA	[30]
CYP17A1	rs4919687	Intronic	Yang et al., 2019	NA	[30]
CYP17A1	rs3781287	Intronic	Yang et al., 2019	NA	[30]
CYP17A1	rs10786712	Intronic	Yang et al., 2019	NA	[30]
CYP17A1	rs6163	Synonymous	Yang et al., 2019	NA	[30]
CYP17A1	rs743572	5’UTR	Yang et al., 2019	NA	[30]
CYP19A1	rs4775936	5’UTR	Yang et al., 2019	NA	[30]
CYP27B1	rs4646536	Intronic	Yang et al., 2019	NA	[30]
CYP2D6	rs35742686	Frameshift	Lopes et al., 2022	Non-Hispanic US	[36]
CYP2D6	*2 (rs16947) (rs1135840)	Missense	Mosley et al., 2018	USA	[37]
CYP2D6	*3 (rs35742686)	Frameshift	Mosley et al., 2018	USA	[37]
CYP2D6	*4 (rs3892097 )	Splicing	Mosley et al., 2018	USA	[37]
CYP2D6	*5	Deletion	Mosley et al., 2018	USA	[37]
CYP2D6	*6 (rs5030655)	Frameshift	Mosley et al., 2018	USA	[37]
CYP2D6	*7 (rs5030867)	Missense	Mosley et al., 2018	USA	[37]
CYP2D6	*8 (rs5030865)	Missense	Mosley et al., 2018	USA	[37]
CYP2D6	*9 (rs5030656)	Deletion	Mosley et al., 2018	USA	[37]
CYP2D6	*10 (rs1065852)	Missense	Mosley et al., 2018; Satkunananthan et al., 2022	USA; Asian	[27,37]
CYP2D6	*11 (rs28399447) (rs28371685)	Missense	Mosley et al., 2018	USA	[37]
CYP2D6	*15 (rs5030867)	Missense	Mosley et al., 2018	USA	[37]
CYP2D6	*17 (rs28371706) (rs16947)	Missense	Mosley et al., 2018	USA	[37]
CYP2D6	*29 (rs61736512) (rs16947) (rs59421388) (rs1135840)	Missense	Mosley et al., 2018	USA	[37]
CYP2D6	*35 (rs769258) (rs1058164) (rs16947) (rs1135840)	Missense	Mosley et al., 2018	USA	[37]
CYP2D6	*41 (rs28371725)	Intronic	Mosley et al., 2018	USA	[37]
EGFL6	rs73633565	Intronic	Lee et al., 2019	European-Americans, Nigeria; Han Chinese; and Japanese	[28]
ELAC2	rs11545302	Synonymous	Yang et al., 2019	NA	[30]
ESR1	rs73625113	Intronic	Rahmioglu et al., 2023	European and East Asian	[38]
ESR1	rs2234693	Upstream	Wang et al., 2013	China	[39]
ESR1	rs9340799	Intronic	Wang et al., 2013	China	[39]
FAAH	rs324420	Missense	Yang et al., 2019	NA	[30]
FAAH	rs1571138	Intronic	Yang et al., 2019	NA	[30]
FAAH	rs3766246	Intronic	Yang et al., 2019	NA	[30]
FAAH	rs4660928	TF binding site	Yang et al., 2019	NA	[30]
HOXA10	rs6970537	Intronic	Rahmioglu et al., 2023	European and East Asian	[38]
HOXC10	rs3803042	Non-coding exon	Rahmioglu et al., 2023	European and East Asian	[38]
IL-10	rs1800871	Upstream	Yang et al., 2019	NA	[30]
IL-10	rs3024505	Intergenic	Yang et al., 2019	NA	[30]
IL-10	rs3024498	3’UTR	Yang et al., 2019	NA	[30]
IL-10	rs3024496	3’UTR	Yang et al., 2019	NA	[30]
IL-10	rs1878672	Intronic	Yang et al., 2019	NA	[30]
IL-10	rs1518111	Upstream	Yang et al., 2019	NA	[30]
IL-10	rs1518110	Upstream	Yang et al., 2019	NA	[30]
IL-10	rs3024491	Intronic	Yang et al., 2019	NA	[30]
IL-13	rs1295686	Intronic	Yang et al., 2019	NA	[30]
IL1R1	rs2110726	3’UTR	Yang et al., 2019	NA	[30]
IL1R2	rs11674595	Intronic	Yang et al., 2019	NA	[30]
IL-6	rs2006984	5’UTR	Yang et al., 2019	NA	[30]
IL-6	rs1800797	Upstream	Crescioli et al. 2022	Italy	[40]
KCNA1	rs4766311	3’UTR	Yang et al., 2019	NA	[30]
KCND2	rs1072198	Intronic	Yang et al., 2019	NA	[30]
KCNJ3	rs12995382	Intronic	Yang et al., 2019	NA	[30]
KCNJ4	rs17641121	Intronic	Yang et al., 2019	NA	[30]
KCNJ6	rs858003	Intronic	Yang et al., 2019	NA	[30]
KCNJ6	rs6517442	Upstream	Elens et al., 2016	Sweden	[41]
KCNK9	rs2542424	Intronic	Yang et al., 2019	NA	[30]
KCNK9	rs2545457	Intronic	Yang et al., 2019	NA	[30]
LINC00629	rs73241342	Intronic	Rahmioglu et al., 2023	European and East Asian	[38]
LNC-LBCS	rs6456259	Intronic	Rahmioglu et al., 2023	European and East Asian	[38]
LTA	rs1799964	Upstream	Yang et al., 2019	NA	[30]
NFKBIA	rs8904	3’UTR	Yang et al., 2019	NA	[30]
NFKBIA	rs2233419	Intronic	Yennurajalingam et al., 2021	USA	[31]
NFKBIA	rs2233417	Intronic	Yennurajalingam et al., 2021	USA	[31]
NFKBIA	rs3138054	Intronic	Yennurajalingam et al., 2021	USA	[31]
NFKBIA	rs1050851	Synonymous	Yennurajalingam et al., 2021	USA	[31]
NF-κB	rs230493	Intronic	Yang et al., 2019	NA	[30]
OPG	rs2073618	Upstream	Yang et al., 2019	NA	[30]
OPRM1	rs79910351 (Arg181Cys)	Missense	Skorpen et al., 2016	European	[42]
OPRM1	rs1799971 (Asn40Asp)	Missense	Bugada, 2020; Hajj et al., 2017; Satkunananthan et al., 2022; Yang et al., 2019	NA; Lebanon; Asian; NA	[27,30,33,43]
OPRM1	rs9479759	Intronic	Yennurajalingam et al., 2021	USA	[31]
OPRM1	rs2003185	Intronic	Yennurajalingam et al., 2021	USA	[31]
OPRM1	rs636433	3’UTR	Yennurajalingam et al., 2021	USA	[31]
P2RX7	rs1718125	Intronic	Satkunananthan et al., 2022	Asian	[27]
P2RY12	rs3732765	Missense	Yang et al., 2019	NA	[30]
P2RY12	rs9859538	Intronic	Yang et al., 2019	NA	[30]
P2RY12	rs17283010	Intronic	Yang et al., 2019	NA	[30]
P2RY12	rs11713504	Intronic	Yang et al., 2019	NA	[30]
P2RY12	rs10935840	Intronic	Yang et al., 2019	NA	[30]
PLAUR	rs4760	Missense	Yang et al., 2019	NA	[30]
PTGS2	rs5275	3’UTR	Yang et al., 2019	NA	[30]
PTGS2	rs20417	Upstream	Lee et al., 2006	USA	[44]
RFFL	rs16970540	3’UTR	Lee et al., 2019	European-Americans, Nigeria; Han Chinese; and Japanese	[28]
RHBDF2	rs12948783	Upstream	Galvan et al., 2011	European	[45]
SPON1	rs13421094	Intergenic	Galvan et al., 2011	European	[45]
SPON1	rs12211463	Intergenic	Galvan et al., 2011	European	[45]
SPON1	rs7757130	Intronic	Galvan et al., 2011	European	[45]
SPON1	rs2473967	Intronic	Galvan et al., 2011	European	[45]
SPON1	rs2884129	Intergenic	Galvan et al., 2011	European	[45]
SPON1	rs7104613	Intronic	Galvan et al., 2011	European	[45]
SYNE1	rs71575922	Intronic	Rahmioglu et al., 2023	European and East Asian	[38]
TNF	rs1800629	Upstream	Yang et al., 2019	NA	[30]
TNF	rs1800610	Intronic	Yang et al., 2019	NA	[30]
TNF	rs1800469	Upstream	Yang et al., 2019	NA	[30]
TNF	rs2241716	Intronic	Yang et al., 2019	NA	[30]
TNF	rs1800629	Upstream	Crescioli et al. 2022	Italy	[40]
UGT2B7	rs7439366	Missense	Satkunananthan et al., 2022	Asian	[27]
UGT2B7	rs7438135	Upstream	Tian et al., 2012	Italy	[46]
VDR	rs11568820	Intronic	Yang et al., 2019	NA	[30]
ZNF235	rs10413396	5’UTR	Galvan et al., 2011	European	[45]
*	rs2369049	Intergenic	Genovese and Mao, 2019	USA	[47]

All variants were checked in dbSNP (NCBI) and Asia Ensembl. * means there is no related gene to this variant because it is located in the intergenic site, and TF refers to transcription factor. Both 5′UTR and 3′UTR mean untranslated regions. NA means not applicable due to reasons such as review articles.

**Table 3:** Essential information about methods of pain relief explored in the reviewed studies.

Study	Tools Used to Collect Pain Information from Patients	Findings	Ref.
Cepeda et al.	CPPR and PRPPR	Pain decreased from initial pain following analgesic administration.	[56]
Musshoff et al.	Hair analysis	Hair analysis can be a valuable and supplementary method for isolating patients who take opioid analgesics for pain relief.	[57]
Dalal and Bruera	Personalized therapy and education of patient/family in decision-making	Cancer-related pain is a multidimensional construct resulting from a complicated combination of physiological, socio-cultural, psychological, behavioral, sensory, and cognitive factors.	[58]
Sarzi-Puttini et al.	Selecting an effective, suitable, personalized analgesic prescription for individuals with chronic pain is feasible	Personalized analgesic prescription will improve compliance, general functioning, and QoL.	[59]
Petersen et al.	Prepared an item bank of 16 pain-measurement items appropriate for CAT	The pain CAT is now available for “experimental” uses by the EORTC.	[61]
Balducci and Dolan	Investigated palliative care for disease in elderly patients	Target planning, symptom control, and caregiver attention are the three foundations of effective palliative care.	[62]
Bhatnagar and Gupta	Integrating cancer pain and symptom management into present pain management	Simultaneously collect information and develop guidelines and suggestions for accurate symptom management across a wide range of patients and diseases to provide a personalized strategy for patient care.	[63]
Arthur et al.	Relationship between ECS-CP characteristics and pain treatment results among outpatients	Neuropathy was a poor predictive factor in the treatment of advanced cancer pain.	[64]
Colvin	Repurposing drugs such as metformin	During oncological treatment, any new therapies adopted must not conflict with the tumoricidal impacts of chemotherapy.	[66]
Vimalnath et al.	Production and investigation of Ce-141 as an effective theragnostic agent for metastatic skeletal lesions	Potential value of 141Ce-DOTMP as a theragnostic component proved for tailored patient treatment of cancer patients.	[67]
Sica et al.	Studied the efficiency of the intrathecal pump in 140 patients	Intrathecal is safer than systemic opioids, which frequently require greater dosages to be efficient leading to the probable major side effects.	[68]
Cuomo et al.	Trolley analgesic model	The employment of personalized therapies with dynamic multimodal methods for pain management found.	[70]
LeBaron et al.	BE-SI-C	BE-SI-C has the ability to track and predict pain while also improving self-efficacy, safety, communication, and QoL in cancer patients.	[72]
Oldenmenger et al.	Examined nine BAT questions, such as “How often do you get breakthrough pain?”	BAT is a legitimate and precise questionnaire that may be used in everyday practice to measure breakthrough pain in Dutch cancer patients.	[74]
Ben-Arye et al.	IO therapy in 815 eligible patients receiving cancer treatment in adjuvant, neo-adjuvant, and palliative care settings.	An initial consultation with an integrative clinician and follow-up visits and receiving adjuvant and neo-adjuvant were the benefits.	[76]
Mao et al.	Electroacupuncture or auricular acupuncture	Electroacupuncture and auricular acupuncture reduced pain.	[77]
Batistaki et al.	Investigated the relationship between BTcP, background cancer pain, and analgesic therapy	A multimodal analgesic approach is proposed.	[81]
Masukawa et al.	Established machine learning models in CPM	They predicted social pain, spiritual pain, and severe signs in terminally suffering cancer patients using text data from electronic healthcare records.	[82]
Reddy et al.	Transition from opioids to levorphanol utilizing an ORR of 8.5	Levorphanol was well tolerated and linked to better pain and symptom management.	[84]
Aziz and Cascella	Peripheral neurolytic blocks	Some forms of painful diseases must be handled by administration of less-invasive analgesic procedures.	[85]
Dalal et al.	Utilized level of pain reduction on a scale from 0 to 10 and median PPG	Regular PPG recording may help with personalized pain management.	[86]

Abbreviation: ref: reference; CPPR: calculated percentage of pain reduction; PRPPR: patient-reported percentage of pain reduction; QoL: quality of Life; CAT: computerized adaptive testing; EORTC: European Organization for Research and Treatment of Cancer; ECS-Cp: Edmonton Classification System for Cancer Pain; BE-SI-C: Behavioral and Environmental Sensing and Intervention for Cancer; BAT: Breakthrough Pain Assessment Tool: IO: integrative oncology; BTcP: breakthrough cancer pain; RR: opioid rotation ratio; PPG: personalized pain goal.

**Table 4:** Important information about genes and drugs involved in cancer pain management.

Study	Genes	Drugs/Methods	Ref.
Galvan et al.	SPON1, RHBDF2, ZNF235	Opioids	[45]
Skorpen et al.	OPRM1	Opioids	[42]
Tverdohleb et al.	CYP2D6 expression	Opioids	[90]
Sivanesan and Gitlin	OPRM1	Tramadol and ziconotide	[91]
Obeng et al.	OPRM1, CYP2D6	Morphine, oxycodone, and hydrocodone	[92]
Haji et al.	OPRM1	Morphine	[43]
Mosley et al.	CYP2D6	Oxycodone	[37]
Yang et al.	OPRM1, COMT, CYP2D6, and ILs	Opioid analgesics	[30]
Nissenbaum et al.	CACNG2	-	[93]
Bortsov et al.	CACNG2	Anti-epileptics	[32]
Lee et al.	RFFL/LIG3, ABCC4/MPR4, EGFL6	-	[28]
Genovese and Mao	COMT	Acupuncture	[47]
Xu et al.	OPRM1, OPRK1, OPRD1, SSTR1, SSTR2, and SSTR5	Herbal drugs	[95]
De Bono et al.	BRCA1, BRCA2, ATM	Olaparib	[97]
Bugada et al.	COMT, OPRM1	Opioids	[33]
Rienzo et al.	PRDM12	-	[98]
Reizine et al.	CYP2D6	Codeine, tramadol, hydrocodone	[99]
Saloman et al.	IL1β, IL6, IL2, TNF, MCP1, IL-4, IL-8, CGRP	Pain biomarkers in serum	[100]
Crescioli et al.	IL6, TNF	Opioids	[40]
Satkunananthan et al.	CYP2D6, OPRM1, COMT, ABCB1	Tramadol	[27]
Wang et al.	IL-6, TNF-α	Oxycodone	[109]
Nishizawa et al.	ANGPT1, SLC2A14	Opioid analgesic	[110]
Li et al.	F13B	Sufentanil	[111]

Ref. refers to the references in the main text.

## Data Availability

All relevant clinical data are presented in this article. The raw data can be made available upon request to the first author.
